# Comparative evaluation of a two-reagent cold stain method with Ziehl-Nelseen method for pulmonary tuberculosis diagnosis

**DOI:** 10.1186/1756-0500-6-323

**Published:** 2013-08-13

**Authors:** Yemane Weldu, Daniel Asrat, Yimtubezinash Woldeamanuel, Aregawi Hailesilasie

**Affiliations:** 1 Department of Microbiology, Immunology and Parasitology, School of Medicine, College of Health Sciences, Addis Ababa University, Addis Ababa, Ethiopia; 2 School of Medicine, College of Medicine and Health Sciences, Debre Markos University, Debre Markos, Amhara, Ethiopia; 3 Department of Medical Laboratory Technology, College of Health Sciences, Mekelle University, Mekelle, Ethiopia

**Keywords:** Gabbet’s methylene blue, AFB microscopy, *PTB*, Cold stain, Zeihl-Neelsen, Ethiopia

## Abstract

**Background:**

Bacteriological examination of sputum is the cornerstone in diagnosis of pulmonary tuberculosis in developing world, which is usually done using a Ziehl-Nelseen (ZN) method. However, due to limited laboratory facilities that can satisfy the procedure, applicability of this procedure appears to be adversely affected in field conditions and at peripheral health institutions. Hence, it has become necessary to look for a procedure which can be used as alternative in such conditions.

In a cross-sectional study, using convenient sampling technique 362 pulmonary tuberculosis suspected patients who attended at Mekelle University Hospital (MUH) between November 2011 and February 2012 were included. After obtaining an informed consent, spot- morning-spot sputum samples were collected from suspected patients. Then a set of duplicate slides, of which one was allocated to a two-reagent cold method (a method of staining which requires carbol fuchsine as a primary stain and Gabbet’s methylene blue both as a decolorizer and counter stain) and the other to the Zeihl-Nelseen method were smeared evenly from representative portion of each specimen using the protocol for duplicate smear preparation. Stained smears were read blindly by two technologists at different occasions. Finally to assure quality, all positive smears and 25% of the negative smears were cross checked by senior experienced examiner.

**Findings:**

Overall concordance between the two methods was 99.7% (kappa (κ) = 0.98; 95%, confidence interval 0.93-1.00), and the observed agreement was statistically significant (p<0.001). When evaluated against Ziehl-Nelseen method, sensitivity and specificity of the two-reagent cold staining method were 95.8% (95% confidence interval 93.7-97.9) and 100% respectively. Positive and negative predictive values of the two-reagent cold staining method were respectively 100% and 99.7%. Positive and negative agreements between the two techniques were respectively 97.9% and 99.9%.

**Conclusion:**

The two-reagent cold staining method was found to be a suitable alternative to the conventional Ziehl-Nelseen method; it was at least as specific as Ziehl-Neelsen method although somewhat less sensitive. However, large scale multicentric studies need to be performed for further evaluation of this cold staining method.

## Findings

### Background

Sputum smear microscopy is the cornerstone in diagnosis of pulmonary tuberculosis in developing world, which is usually done using Ziehl–Nelseen (ZN) method [1-3]. However, due to limited Laboratory facilities that can satisfy the procedure, applicability of this procedure appears to be adversely affected in field conditions and at peripheral health institutions [4]. Even in the presence of laboratory facilities it is cumbersome and not suitable in settings with high workloads; it is a multi-stage staining procedure which requires separate steps for de-colorization and counterstaining, application of heat for fixation of the prepared smear and during the staining procedure [2,4]. Recently WHO endorsed XpertMTB RIF assay (company) as a front-line testing for TB and were possible to replace smear microscopy. However, this recommendation is less implemented in most of the resource limited countries due to the fact that its cost is less affordable. Therefore, replacing smear microscopy as the first test for TB is not likely to happen in the near future. Consequently, especially in developing countries with a large number of cases and financial constraints, there is an urgent need for rapid, sensitive and low-cost method for the detection of *Mycobacterium tuberculosis* in clinical samples [5]. Operational research to improve smear microscopy may be the most feasible alternative for better TB diagnosis. Here we evaluate CS (Cold staining) as an alternative test to ZN. CS is one modification of ZN which requires carbol fuchsine as a primary stain and Gabbet’s methylene blue, both as a decolorizer and counter stain. Compared to ZN; CS is simple and easy to learn and practice, it is economical and less cumbersome, is suitable under field conditions, and can be practiced even in remote areas and at periphery where laboratory facilities are limited. There are also more practical advantages of CS like, no need of heating in the staining procedure and eliminated the need for separate decolorizing step (requires only two reagents in the staining procedure) [4]. Therefore, applying of this cold staining technique in developing countries like Ethiopia can have the potential to enlarge the number of AFB (Acid Fast Bacilli) diagnostic services, and by so doing there may be better achievement of case detection rate of smear positive PTB (Pulmonary Tuberculosis) [6].

Although studies, with promising findings, are available in some developing countries such as India and Nepal, all had tried to evaluate the CS for detection of AFB from a single sputum specimen only. Hence, to strengthen the previous findings, the present study aimed to evaluate this cold staining method in spot-morning-spot sputum specimens which is the conventional microscopy case finding strategy.

## Methods

### Sputum samples and study population

After obtaining an informed consent, a total of 1086 sputum samples collected from 362 pulmonary tuberculosis suspected patients attending the hospital were used in the study; spot-morning-spot specimen per patient.

### Smear preparation

To obtain representative smear the following steps were carried out: first two frosted end slides were prepared, and then from the purulent part of sputum sample was extracted and placed on the center of one of the slides using a sterile cotton swab. By holding each slide by its frosted end the second slide was placed on top of the first slide, and then tried to move the slides against each other in several directions with a rotating motion. Finally, slides were tried to separate by pulling away from each other horizontally. If the specimen is not evenly spread on both the smears, the process may be repeated again [7,8].

### Design

A cross-sectional study was conducted from November 2011 to February 2012 at Mekelle University Hospital, Tigray, Ethiopia, in which convenient sampling technique was applied to obtain sputum samples from the suspected patients. From the set of duplicate smears, one was allocated to the Zeihl-Nelseen method and the other to the two-reagent cold staining method. Slides were coded, thereby ruled out selective bias. Stained smears were read blindly by two technologists independently; during microscopic examination all examiners had no information about the result of other examiner. To assure quality, all positive smears and 25% of the negative smears were cross checked by senior experienced examiner.

## Z-N Method

The Z-N stain was carried out following NTCP (National Tuberculosis Control Program) guidelines. Prepared sputum smears were flooded with filtered 1% carbol fuchsine and heated until steaming but not boiling for 5 minutes. After slides had been rinsed with tap water, 3% acid alcohol was used to decolorize smears for 3 minutes. Then slides were rinsed and counterstained with 0.1% methylene blue for 1 minute. Finally, the slides were washed, air-dried and examined using an oil immersion objective. The composition of the reagents was as follows: 1% carbol fuchsine: basic fuchsine 10 g, molten phenol 50 ml, 95% ethanol 100 ml, distilled water 850 ml; 3% acid alcohol: 96% ethanol 970 ml, hydrochloric acid 30 ml; 0.1% methylene blue: methylene blue 1 g, distilled water 1000 ml [9].

## Cold method

The cold method was performed as follows; briefly, prepared smears were flooded with reagent I (1% carbol fuchsine) and allowed to stand at room temperature for 10 minutes, then after washing with tap water they were decolourised and counterstained with reagent II (Gabbet’s methylene blue) for another 2 minutes. Finally, slides were washed, air-dried and examined using an oil immersion objective. The contents of the reagents are as follows: reagent I (1% carbol fuchsine): basic fuchsine 10 g, molten phenol 50 ml, 95% ethanol 100 ml, distilled water 850 ml; reagent II (Gabbet’s methylene blue): methylene blue 10 g, 95% ethanol 300 ml, 97% sulphuric acid 200 ml, distilled water 500 ml [1,4].

### Statistical analysis

Data were recorded and analyzed using SPSS version 16.0 software. The agreement between Z-N and Cold Stain Method was based on Kappa (к) value; agreement was considered as significant if p< 0.05. Sensitivity, specificity and predictive values of the cold stain were also calculated.

The protocol was approved by the ethics committee of Microbiology, Immunology and Parasitology Department, School of Medicine, College of Health Sciences, Addis Ababa University.

## Results

### Study population

A total of 362 patients with suspected case of pulmonary tuberculosis were studied. Of the total, 24 (6.6%) had proven tuberculosis by positive AFB microscopy using the ZN method. On the other hand, among the 362 patients with suspected case of pulmonary tuberculosis, 23 (6.4%) were positive for AFB by the cold staining method. Using ZN and CS methods AFB detection and grading in the Spot-Moring-Spot sputum specimen is shown in Table 1 and Table 2, respectively.

**Table 1:** AFB detection and grading in spot-morning-spot sputum specimens using Z-N method

**ZN; no. (%)/362**						
***Sputum specimen***	**Grade**					
	**3+**	**2+**	**1+**	**Scanty**	**Negative**	**Total**
1st Spot	8(2.2)	8(2.2)	6(1.7)	2(0.6)	338(93.4)	362(100)
Moring	9(2.5)	7(1.9)	6(1.7)	2(0.6)	338(93.4)	362(100)
2nd Spot	7(1.9)	10(2.8)	5(1.4)	1(0.3)	339(93.6)	362(100)

*ZN* Ziehl-Neelsen, *AFB* acid fast bacilli; 3+ = More than 10 AFB per oil immersion field in at least 20 fields; 2+ = 1–9 AFB per oil immersion field in at least 50 fields; 1+ = 10–99 AFB in 100 oil immersion fields; Scanty = 1–9 AFB in 100 oil immersion fields.

**Table 2:** AFB detection and grading in spot-morning-spot sputum specimens using CS method

**CS; no. (%)/362**						
***Sputum specimen***	**Grade**					
	**3+**	**2+**	**1+**	**Scanty**	**Negative**	**Total**
1st Spot	5(1.4)	11(3.0)	6(1.7)	1(0.3)	339(93.6)	362(100)
Moring	7(1.9)	10(2.8)	5(1.4)	1(0.3)	339(93.6)	362(100)
2nd Spot	6(1.7)	9(2.5)	7(1.9)	1(0.3)	339(93.6)	362(100)

*CS* Cold stain.

### Diagnostic validity of CS using ZN as a reference method

Overall concordance between the two staining methods was found to be 99.7% (Kappa (κ) = 0.98), where positive and negative agreements were respectively 97.9% and 99.9%. When evaluated against ZN method sensitivity, specificity, positive and negative predictive values of the two- reagent cold staining method were 95.8%, 100%, 100% and 99.7%, respectively (See Table 3 below). For the two techniques, comparative result for spot-morning-spot sputum specimens is shown below.

a) First-spot sputum specimen

Of the total 362 specimen, 4 (1.1%) samples were reported differently by the two methods; 3 samples graded as 3+ by Z-N were read as 2+ by the CS and 1 sample reported scanty by Z-N was negative by the cold stain method (See Table 4).

b) Morning sputum specimen

Of the 362 morning sputum specimens, 4 (1.1%) specimens graded as 1+ (2 samples), 3+ (1 sample) and 3+ (1 sample) by the Z-N were read as 2+, 1+ and 2+, respectively by the cold stain method (See Table 5).

c) Second-spot sputum specimen

Of the 362 samples, 5 (1.4%) specimens which were 2+ (2 samples), 2+ (1 sample) and 3+ (2 samples) by the Z-N method were read respectively as 1+, 3+ and 2+ by the two-reagent cold staining method (See Table 6).

**Table 3:** Overall comparison of cold stain method with the Ziehl-Neelsen method

**ZN; no. (%)**			
**Report**	**Positive**	**Negative**	**Total**
Positive CS	23(6.4)	0(0)	23(6.4)
Negative	1(0.3)	338(93.4)	339(93.6)
Total	24(6.6)	338(93.4)	362(100)

*ZN* Ziehl-Neelsen, *CS* Cold stain; Positive = any positive (scanty, 1+, 2+ and 3+); Negative = no Acid fast bacilli per oil immersion in at least 100 fields; Sensitivity = 95.8%; Specificity = 100%; Positive predictive value =100%; Negative predictive value = 99.7%; Agreement = 99.7; Kappa (κ) = 0.98.

**Table 4:** Comparison of ZN and CS methods for detection and grading of AFB in first-spot sputum specimens

**ZN; no. (%)/24**							
	***Grade***	**3+**	**2+**	**1+**	**Scanty**	**Negative**	**Total**
	3+	5(1.4)	0(0)	0(0)	0(0)	0(0)	5(1.4)
	2+	3(0.8)	8(2.2)	0(0)	0(0)	0(0)	11(3.0)
CS/23	1+	0(0)	0(0)	6(1.7)	0(0)	0(0)	6(1.7)
	Scanty	0(0)	0(0)	0(0)	1(0.3)	0(0)	1(0.3)
	Negative	0(0)	0(0)	0(0)	1(0.3)	338(93.4)	339(93.6
	Total	8(2.2)	8(2.2)	6(1.7)	2(0.6)	338(93.4)	362(100)

*AFB* acid fast bacilli; 3+ = More than 10 AFB per oil immersion field in at least 20 fields; 2+ = 1–9 AFB per oil immersion field in at least 50 fields; 1+ = 10–99 AFB in 100 oil immersion fields; Scanty = 1–9 AFB in 100 oil immersion fields.

**Table 5:** Comparison of ZN and CS methods for detection and grading of AFB in morning sputum specimens

**ZN; no. (%)/24**							
	***Grade***	**3+**	**2+**	**1+**	**Scanty**	**Negative**	**Total**
	3+	7(1.9)	0(0)	0(0)	0(0)	0(0)	7(1.9)
	2+	1(0.3)	7(1.9)	2(0.6)	0(0)	0(0)	10(2.8)
CS/23	1+	1(0.3)	0(0)	4(1.1)	0(0)	0(0)	5(1.4)
	Scanty	0(0)	0(0)	0(0)	1(0.3)	0(0)	1(0.3)
	Negative	0(0)	0(0)	0(0)	1(0.3)	338(93.4)	339(93.6)
	Total	9(2.5)	7(1.9)	6(1.7)	2(0.6)	338(93.4)	362(100)

**Table 6:** Comparison of ZN and CS methods for detection and grading of AFB in second-spot sputum specimens

**ZN; no. (%)/23**							
	***Grade***	**3+**	**2+**	**1+**	**Scanty**	**Negative**	**Total**
	3+	5(1.4)	1(0.3)	0(0)	0(0)	0(0)	6(1.7)
	2+	2(0.6)	7(1.9)	0(0)	0(0)	0(0)	9(2.5)
CS/23	1+	0(0)	2(0.6)	5(1.4)	0(0)	0(0)	7(1.9)
	Scanty	0(0)	0(0)	0(0)	1(0.3)	0(0)	1(0.3)
	Negative	0(0)	0(0)	0(0)	0(0)	339(93.6)	339(93.6)
	Total	7(1.9)	10(2.8)	5(1.4)	1(0.3)	339(93.6)	362(100)

## Discussion

Applicability of the Ziehl-Nelseen technique appears to be adversely affected, especially at peripheral health institutions, due to limited laboratory facilities that can satisfy the procedure. Hence, the present study aimed to evaluate CS method which can be used as alternative in such conditions. In the present study, the interesting finding is that the CS Method has not shown any positive result even on a single specimen which was not positive by the ZN method. On the other hand, there was one study participant negative for AFB by CS method only. These two observations suggest that CS Method is at least as specific as ZN Method although somewhat less sensitive, i.e. the cold staining was found to be 100% confident in detecting positive results by this specific test, to be truly positive also by the conventional Z-N staining method, and it was with 99.7% confidence in ruling out pulmonary tuberculosis, if used instead of the Z-N staining technique (See Table 3).

In interpretation of results, the overall concordance between the two staining methods evaluated here was verygood [10] (Kappa (κ) = 0.98), and the observed agreement was found to be also statistically significant (p < 0.001). Therefore, in the present comparative evaluation study, the assessments made for these two staining techniques were not random.

Yield of positive result by ZN and CS was found to be comparable with each other (6.6% vs. 6.4%), which is in agreement with the reports of other scholars: 24.5% vs. 24.3% (Chandrasekaran et al.*,* 1991) [3]; 20.0% vs. 18.6% (Pandey et al., 2009) [11]; 14.3% vs. 13.4% (Gokhale et al., 1990) [4]; 7.9% vs. 7.1% (Gupta et al., 2009) [2]; 14.2% vs. 14.4% (Shrestha et al., 2005) [5]; 38.5% vs. 39.8% (Selvakumar et al., 2002) [12]; and 32.1% vs. 32.7% (Vasanthakumari et al., 1986) [1].

Possible explanations for the slight difference in yield of results by the ZN and cold stain could be defined as follows: in the present study, generally, in microscopic examination of smear slides prepared by the cold staining method, it was observed that AFB organisms appeared more delicate, fainter (which is closer to their natural morphology) and less brighter against background than those seen with the Z-N stain, which may be the reason for the false negative results compared with by the Z-N method. Whereas, in Z-N method since there is a better penetration of stain through the complex cell surface structure due to heating effect, organisms appeared brighter against background though there is slight change in morphology. Although it is difficult to conclude, since none of the specimens could be cultured for want of facilities, the ZN could give a few false positive results. Similar observation was seen by other scholars too (Gokhale et al.*,* 1990 [4], Pandey et al., 2009 [11]).

Distribution of bacilli with in a sputum sample may get varied and this could be also one reason for the difference in reporting results. For example, as we can see the grading results from Tables 4, 5 and 6, in the sputum sample which was missed by the cold staining method, the AFB smear showed lower concentration of bacilli by the ZN (that is scoring: scanty, scanty and negative) for the spot-morning-spot sputum specimen respectively. Therefore, this difference in report of the ZN and CS suggests that findings could be affected by any one of the above mentioned reasons.

### Limitations

Due to limited resources, none of the specimens could be confirmed by culture or other molecular techniques. The design also lacked follow-up of cases with discordant results.

## Conclusion

In conclusion, the two-step cold staining method was found to be a suitable alternative to the conventional Z-N method for microscopic examination of pulmonary tuberculosis. There was very-good agreement (κ = 0.98) between the two techniques for the diagnosis of pulmonary tuberculosis. The observed agreement was also statistically significant (p< 0.001). The Cold Staining Method was at least as specific as ZN Method although somewhat less sensitive (100% and 95.8% respectively). Positive and negative predictive values of the Cold Staining Method were respectively 100% and 99.7%. Overall agreement between the two techniques was 99.7%, and positive and negative agreements were respectively 97.9% and 99.9%. However, large scale multicentric studies need to be performed for further evaluation of this Cold Staining Method.

## Abbreviations

AFB: Acid fast bacilli; CS: Cold stain; NTCP: National tuberculosis control program; PTB: Pulmonary Tuberculosis; SPSS: Statistical package for social science; TB: Tuberculosis; ZN: Ziehl Neelsen.

## Competing interests

The authors declared that there were no personal and organizational competing interests.

## Authors’ contributions

YW: Conception and initiation of the study, design, implementation, analysis and interpretation of data, and writing of the thesis work. DA: Design, implementation, guidance, revising for intellectual content and co-writing of the thesis work. YW: Design, implementation, revising for intellectual content and co-writing of the thesis work. AH: Guidance, comments and unreserved support in facilitating good working lab environment. All authors read and approved the final manuscript.

## Authors’ information

Yemane Weldu is currently an instructor at Debre Markos University with a back ground of Medical Laboratory Technology (BSc), and Master of Science in Medical Microbiology (MSc).

Daniel Asrat currently works at Addis Ababa University as an instructor and his academic status is medical doctor (MD), then Master of Science and post doctoral in Medical Microbiology (MSc, PhD).

Yimtubezinash Woldeamanuel currently works at Addis Ababa University as an instructor and her academic status is medical doctor (MD), then Master of Science and post doctoral in Medical Microbiology (MSc, PhD).

Aregawi Hailesilasie currently works at Mekelle Universtry Hospital as Laboratory Technologist, and he has a bachelor of degree in Medical Laboratory Technology (BSc).
